# Dental Spiritualism

**Published:** 1886-02

**Authors:** Louis Ottofy

**Affiliations:** Chicago


### THE DENTAL REGISTER.

VOL. XL.] FEBRUARY, 1886. [No. 2.

### ORIGINAL COMMUNICATIONS.

### Dental Spiritualism.

LOUIS OTTOFY, D. D. S., CHICAGO.

The vastness of our theme hardly admits it to be as fully treated in a single paper, as we should desire, because it forces the discussion of the undecided and indefinite questions of "vital force," "magnetism," "mental force," "electricity," "nervous force," the circulation of the blood and other questions which at first seem remote to the subject. There might be some objection to the use of the word "spiritualism," in a strictly scientific essay, and to clear away any doubt as to its propriety and at the same time explain its meaning, I quote from Prof. Elliott Coues: "No scientist who acknowledges the validity of the science of psychology, and no philosopher who recognizes the validity of abstract ideas, objects to the word mind." I must therefore be permitted to speak of spirit, or 'soul,' if you please, as something which, like mind is a legitimate subject of inquiry: first, as to whether it exists or no; second, if it exists, whether it be of protoplasmic nature or no; third, if it be not that product of the aggregation of matter, what sort of a product it may be, for I consider this inquiry especially pertinent to any discussion of life. Our alternative, you know, is, that all vital phenomena, all manifestations whatsoever of life, are to be counted among the accomplishments of protoplasm, or are to be otherwise accounted for.

"Much difference of opinion as to the reality of 'soul' might be reconciled if disputants could catch each other's meaning and agree upon a definition of the term. But this is very difficult though we all know what is meant when a human soul is in mention. Many deny there is any such thing; many waive the question, neither affirming nor denying; most ascribe a soul to man alone; some concede a soul to every atom of inorganic matter as well as organized bodies. My view defines soul as the quantity of spirit which any living being may or does possess at any time. But this requires a definition of "spirit," from which all conceptions of matter are not absolutely excluded. Spirit is nothing if not immaterial; force is likewise immaterial, but I think all persons recognize a distinction between spirit and any mechanical force, such as gravitation. My mind affords no definition of spirit, if I may not call it *self-conscious force.* Self-conscious force being illimitable in time and space, and its sum being, in a word, infinite, I am unable to draw any distinction between spirit in its totality and that Universal Mind or Supreme Intelligence, which we mean when we speak or think of God."

The question of the existence of forces in the human body, whose exact mode of action is not fully understood, is an open and disputable one. No one present, doubts the assertion that what are known as nervous, mental, and vital-force, are not yet entirely understood by the physiologist, psychologist or philosopher, and yet that such forces do exist, and that they form an active part in our every day life, is not questioned. Even the most pronounced sceptics of the present day admit that there is a something in the Universe, call it by any name, which is something more than human.

Whether thought and its accompanying peculiar phenomena, are simply molecular changes, and take place without the presence and influence of any force outside of the simple chemical combustion of cells, is not satisfactorily established. Indeed, that the circulation of the blood is by no means fully explained by physiologists, is equally true. Thus it seems that some functions which take place within our body, are not to be explained by either the advanced position of chemical or physiological science, forces are at work which do not allow themselves to be analyzed the same as other actions with which we are familiar. In fact, this condition clearly tends to indicate the existence of some power within the body which is not a portion or parcel thereof. The law of the convertibility of force into matter and *vice versa*; and the indestructibility of force and matter being unquestioned, it is doubtful how any thinking person can entertain the belief, that all the functions of the body cease with death, and all is ended. It is true that the chemical elements return to their original condition, but as force can not be destroyed, what becomes of the peculiar vital and mental phenomena? These conditions of facts are leading the most sceptical thinkers, to the admission, that there is somethiug within the body, a spirit, a soul, a part of the macrocosm, if you please, which has not yet placed itself at the disposal of man's investigative inquiry, and which by the inevitable laws of God, remains sealed to mortal man.

DeWette the great learned rational Universal doubter in Germany admitted amid the "sneers of the acutest school of rationalism of which he was the leader," the existence of something beyond materialism. Evolutionists and sceptics, have within recent years placed more flexibility on former expressions, and even agnostics are gradually falling from their creed. Science and knowledge yield reluctantly to the inevitable truth.

An infinite existence certainly permeates all things which we denominate as living, and in the course of nature, that force is everlasting. There is no question that every living cell, from the simplest form of organization to the most complex, contains within it in a condensed spiritual form, its full capabilities, its every possible reproductive power is there outlined, and by its very essence it is capable of filling space in any scale from the micro to the macrocosmic. In this connection the well-known fact may be mentioned, that breeders can reproduce a certain color or spot in an offspring, by exposing to the view of the parents during coition an animal of that color, or with that particular spot, how is that color transmitted? The spermatozoid carries stamped within its microcosmic compass the full spiritual form not only of the future being but of all generations therefrom to issue. There is no doubt but that "life" can exist without being observable by our senses, as it would invariably have to be in a material form for that purpose, no material substance, containing " life," however, can exist without a spiritual outline. That such a form which may be designated ethereal or spiritual, does exist, is borne out by numerous invincible facts, especially by the laws of physical reproduction and heredity. In cases where bodily injury is perpetrated, nature reproduces or endeavors to reproduce the parts in full conformity with the existing outline, excepting in such cases where the injured parts are too complicated. Illustrations in support of this fact are numerous, especially so among the lower forms of life, as the crustaceans; in the tadpole the tail or entire leg are reproduced. Amputated human limbs or other complex parts are not reproduced because of their complexity, but the existence of their spiritual outline, can be hardly questioned; it is well-known that persons who have suffered amputation, often complain of feeling pain in the toes or fingers of the amputated member, and also, that they are capable of moving them, but of course, can perform no action with them. It is not infrequent that pain is felt in the *spaces* (not the sockets) where teeth formerly were; artificial teeth are also known to "ache." The established laws of heredity furnish very satisfactory proofs of the existence of a spiritual outline (in a condensed or compressed form) in the spermatozoid, or ovum, or both; and one of its convincing proofs is well illustrated, in the cases of six-toed or six-fingered persons, where successive amputations, in successive generations, of the supernumary organ have failed to eradicate it in the offspring. A few years ago I saw somewhere expounded the theory, that by extracting in every person all the second bicuspids, in course of time those teeth would not be reproduced, and that thus the overcrowding of the teeth will be averted, but the foregoing practical illustrations prove the fallacy of that idea.

That the cells of the brain should within their physical selves contain the power by which its marvellous results are produced, that all forces originating from that central nervous organ should take place on the same principle as all other productions of force, namely by the combustion of cells only, is certainly very much in question, and the fervency of such an advocacy is mainly due to a formerly very prevalent sceptical notion of materialization, it having been considered unscientific to entertain *any* views, unless they can be demonstrated to the senses by known arts. However, the fact that such a substance as hydrogen or oxygen exists, is not questioned, although neither has ever been observed by any of the senses. We judge of their existence only by their actions on other substances, which we know to exist; and so we judge of spiritual existence by its manifestations. That such a substance as a vacuum exists, or such a condition, if you please, is not doubted, and yet no sense has ever perceived it; the word "substance" is proper when speaking of a vacuum, as a space containing absolutely nothing is inconceivable in the Universe.

One other remarkable proof, which can only be referred to in this paper, of the existence of some unanalyzed power in the body is furnished by the wonderful process, which has baffled physiologists from Harvey's exposition to the present day, the circulation of the blood; it is a remarkable fact that there is no physiological work extant, which explains to the full satisfaction of the writer, sceptic or otherwise, that all the forces of the known body which are, or could be utilized, are sufficient to perform that process, if considered from a physical, mechanical or chemical standpoint; the propelling capacity of the left ventricle of the heart, the contractility of the coats of the arteries, the so-called suction force of the veins and the right ventricle, gravity, chemical affinity, capillary attraction, muscular compression upon the conduits, the affinity of the blood for oxygen in the lungs, and of the tissues for the same in the body, the siphon principle, or the influence of nervous force, etc., all, and all other forces which are known to assist or which are supposed to assist in performing it, are insufficient to complete it. Although some of them are very essential assistants, nevertheless the force of the "vital principle" is *the* primary power. All forces live, as such or as matter in one form or another, and as the circulation of the blood is carried on (in addition to known physical, mechanical and chemical forces) by another unexplainable force, that force lives on forever. By the withdrawal of that one activity (life), the known forces which perform the functions of the body are altered, that withdrawn force, whether it be vital, mental or spiritual, exists in some form and somewhere, and indeed, it is but natural that the problem of life remain unsolved, its solution is the end of life in the capacity and form in which we understand life, or in other words to learn life, death must ensue; the familiar words "in the midst of life we are in death" are equally true when transposed: "In the midst of death we are in life."

Animal magnetism, personal attraction and repulsion, are certainly forces or conditions which come more or less under the observation of the dentist. There are not many practitioners who have made an effort to investigate the subject, but will readily admit that there is "something in it;" and there is no reason why this power should not be cultivated and used by the dentist to the extent at least, of partial anaesthesia during short operations of a painful nature. There are unquestionable men who can control and direct such an amount of magnetism into the body of a patient, as to insure insensibility to pain, that has been done; and that this sustaining power is not due solely to a previous preparation of the mind to receive the impression, that no pain would result, which might be the case to some extent, if a person was told that he is about to be magnetized, and that in consequence thereof no pain would be felt during the operation, is based on the fact that the hands were simply laid upon the temples (the patient being informed) for the purpose of placing the head in the proper position and that the patient may become quieted. There are a large number of "nervous" persons who could be supplied by the majority of dentists, without very much loss to themselves, with a large amount of spiritual sustenance, which would help them to bear pain with more ease.

"In nervous persons the condition is a want of *nerve force* not a super supply, such persons when in the chair and being operated on do draw on the *sympathetic* operator for a re-supply of nerve force to enable them to bear the operation. Some men whose own supply of nerve force is too small even for their personal wants may by greatly sympathizing so impart of their nerve force as to greatly benefit the patient, but always at their own expense, so that after a hard day's operating for such a patient as here described, they will be in a very nervous condition, that is, nerve force has been imparted till there is a decided want felt. Take a lady who is all used up with uterine displacements, backache, feeble health, no courage to bear pain, in other words as nervous as she can be. *If you* will dampen your hands just a little and put them on her face *and forehead* for a few moments you will feel the loss of a something that you will say. 'I perceive that virtue (or force) has gone out of me.'"

"I think direct contact is essential to this. In your operating for a robust healthy person you will perceive the difference very sensibly. Strong, robust, unsympathetic dentists do not freely give of their nerve force, the patient will soon distinguish the difference between such an operator and the one who, in their make-up is more like themselves; with a ready sympathy, a free imparting of their nerve force. They are very prone to patronize the latter rather than the former, so that if you will notice such operators have that class of patrons in a greater proportion than strong robust dentists.*

There is no question in my mind but that nearly every patient is "influenced" more or less by the operator or *vice versa*, so for instance in the latter part of an exhausting day's labor at the chair a nervously powerful patient can supply sufficient spiritual power to the operator for his use to sustain himself without causing undue fatigue. Again, when both operator and patient are fully aware of a certain amount of pain which is to be inflicted to accomplish a certain operation, the patient, if spiritualy so constituted, can "give off" sufficient power to not only assist the operator, but sustain himself as well without the fatigue to one and pain to the other, which otherwise would be the case. Dentists, especially such, who have among their patients a large number of the puny, malformed products of the higher class, those whose systematic functions have been chronically impaired by over-study, lack of exercise, or faulty exercise in excess, and such who live in feverish, malarial and other districts where malignant diseases are prevalent, should cultivate the power of magnetic or

*J. F. Sanborn, M.D., D.D.S.

spiritual influence over their patients. To be able to "give a piece of one's mind" is very near the truth but is not grammatically correct, but to give one a piece of his spirit (which itself is an ethereal substance) is proper and capable of performance, and this is the secret of the success of a great many dentists. We among ourselves understand that influence by another name; we call it sympathy. Sympathy is nothing if it is not something which is capable of being imparted, and made part of the personality of another, and if it is such, it is a something which from its nature may properly be called soul force or spirit. Nay more, the expression, "the heart goes out in sympathy" is as true as it is beautiful, for it is known that very sympathetic persons feel that a something from their innermost selves draws toward the object of their sympathy.

This feeling of sympathy, then, is well worthy the cultivation of every dentist who treasures the welfare of his patients and it is but meet that we should be as humane as to take advantage of all forces, of all known appliances or effects to alleviate as much as possible, existing pain, and inflict the minimum. It would cause surprise to listen to the experience of patients who have been under the care of several operators; making due allowance for temporary causes of difference in susceptibility for pain, what a difference there is in a sympathetic treatment.

There are some essentials in the exercise of this function of spiritual sympathy which we should bear in mind when desiring to diminish the suffering of a patient from existing pain, from pain inflicted by us in order to make our manifestations effective; the mere cold-hearted expression, "I am very sorry to hurt you so much," while a bland, icy smile is playing over our features don't help the patient very much to bear the pain; our professions, if is necessary to make them by words, must be inspired by sincerity, truth and understanding; our actions must depict composure and steadiness. The patient should be able to read in our features that we understand his case and that our professions of sympathy are sincere, let our words (when speaking of pain) be truthful in every particular. Let us not suppress the fact that pain must be consequent upon our actions, there is no harm, however, in measuring and stating the probable extent of pain. A steady, composed bearing should always exist, as thus the confidence of the patient soon wins the sympathy which the face expresses or should express.

There is unquestionably, however, a class of persons who are either spiritually indifferent or negative, and over whom, therefore, no influence can be exerted; and in consequence of this belief the hypothesis exists, that persons are electrically charged either positively or negatively and that therefore affinity or repulsion exists among all persons; just to what degree this is true, we are unable to state, but all have observed the fact of the existence to a certain degree of such a condition. In cases of the kind where these pre-existing conditions are unfavorable, the dentist should exert as much will power as possible to overcome the existing antipathy, and this can be done in most cases, with good success; there are persons, however, in whom the opposition to any influence is so marked, that both the patient and operator are obliged to suffer pain or fatigue, unavoidably. These are generally patients who soon seek another dentist.

Mesmeric influence is another but similar exhibition of spiritual power and consists of the ability of one person to transmit a portion of its spiritual substance—individuality, if you please, to another, in such a degree that pain may be produced or prevented at the option of the transmittor; those who are fortunate in possessing this power, undoubtedly exert a great deal of influence over their patients, especially is this true in operations of a painful nature and short duration, where by bodily contact the operator imbues the body of the patient for a short time with his own spirituality to such a degree that the feeling of pain is diminished. The physical body in its normal condition with the spiritual body also in its normal condition are so equalized that when both work in natural harmony, injury to any part of the sensibly known body is communicated by wires of transmission to the spiritual individual; that is, pain is a product capable of transmission the same as electricity, and when both bodies are normal the pain is felt, but if there is a super supply of spiritual substance, the pain is neutralized by that super supply of vital force and no pain is the result. The law of the correlation of forces is therefore true, not only so far as it is applied to physical bodies, but of spiritual bodies. The extinguishing of the pain then, is not annihilation but only transmission into another form, namely, into spiritual loss of the operator which is by him, if healthy, not perceived as much as the pain would have been by the patient because it is of a different nature, although the same in quantity. The same is the difference between worry and thought; and on the same principle that no force is lost, the illustration is familliar that a brick raised fifty feet retains just so much potential force as it required to raise it to that height, it can exert that force by falling; the movement of the hour and minute hand and the friction of the wheels of the mechanism of a time-piece exactly equals the force exerted in winding the spring. Mesmerism has been known and applied extensively, especially on the Continent, in minor surgical operations including the extraction of teeth, in which latter operation it will be more extensively applied when mesmeric influence is more thoroughly and independently investigated and its true virtues made better known.

Though not strictly within the province of this paper perhaps it will not be entirely out of place to notice one other fact, namely, that although the Scriptures contain fifty-three different verses bearing upon the teeth, (a number of those are mere repetitions, nearly all of them use the teeth merely for purposes of illustration the same as the eyes or hands. Job is credited with saying: " I escaped with the skin of my teeth," tbis sentence, if the literal meaning of the original Hebrew is correctly translated, expresses a truth, which only the present century demonstrated, Nasmyth having discovered the dental cuticule a thin epithelial membrane covering the enamel of the teeth and which is a continuation of the gum and the lining of the socket of the tooth, and in every sense appropriately called a skin, there is no question of its retention upon the teeth for some time after eruption, and indeed, it is but possible, that it may remain a lifetime.

